# Weighted gene co expression network analysis (WGCNA) with key pathways and hub‐genes related to micro RNAs in ischemic stroke

**DOI:** 10.1049/syb2.12016

**Published:** 2021-04-20

**Authors:** Xiang Qu, Shuang Wu, Jinggui Gao, Zhenxiu Qin, Zhenhua Zhou, Jingli Liu

**Affiliations:** ^1^ Department of Neurology The First Affiliated Hospital of Guangxi Medical University Shuangyong Road Nanning Guangxi China

## Abstract

Ischemic stroke (IS) is one of the major causes of death and disability worldwide. However, the specific mechanism of gene interplay and the biological function in IS are not clear. Therefore, more research into IS is necessary. Dataset GSE110993 including 20 ischemic stroke (IS) and 20 control specimens are used to establish both groups and the raw RNA‐seq data were analysed. Weighted gene co‐expression network analysis (WGCNA) was used to screen the key micro‐RNA modules. The centrality of key genes were determined by module membership (mm) and gene significance (GS). The key pathways were identified by enrichment analysis with Kyoto Protocol Gene and Genome Encyclopedia (KEGG), and the key genes were validated by protein‐protein interactions network. Result: Upon investigation, 1185 up‐ and down‐regulated genes were gathered and distributed into three modules in response to their degree of correlation to clinical traits of IS, among which the turquoise module show a trait‐correlation of 0.77. The top 140 genes were further identified by GS and MM. KEGG analysis showed two pathways may evolve in the progress of IS. Discussion: CXCL12 and EIF2a may be important biomarkers for the accurate diagnosis and treatment in IS.

## 1 BACKGROUND

Ischemic stroke (IS) is the second leading cause of mortality and third cause of disability in the world. The incidence rate of IS was over 16 million 900 thousand cases in 2010 and it was about 16 million 900 thousand globally [1]. According to up‐to‐date statistics, 1.12 million adult patients over 20 died of stroke in China. Pathological studies have confirmed that the main causes of ischemic stroke are the formation of atherothrombosis and cardiac embolism [2]. At present, the conventional treatment strategy is to perform rapid mechanical and chemical thrombolysis after stroke. These treatment requires instant therapy and intervention, besides increased thrombolysis is also related with the high risk of bleeding (including intracranial hemorrhage) [3]. Therefore, rapid detection with serum biomarkers to detect the ischemic stroke (IS) event may be of important clinical value for disease prevention and treatment, intervention and prognosis. At present, the diagnosis of IS mainly depends on cardiac tomography imaging, such as magnetic resonance imaging (MRI) [4] and computed tomography [5], which is labor‐redundant and time‐consumptive. Thus it is of great significance to find potential molecular biomarkers for the prevention, diagnosis and treatment of IS.

Myocardial infarction is usually diagnosed by ECG,recent study inferred circulating RNAs may also be valuable for the diagnosis of acute ischemic stroke. MicroRNAs (miRNAs) are small RNA molecules, which are about 22 nt sequences that have an important role in the transcription regulation and degradation of mRNA. Because of the feasibility of detection and stability in blood samples, they can be used as valuable biomarkers [6]. Many studies have confirmed the expression of miRNA in patients with acute ischemic stroke, but as a biomarker, the potential and mechanism of miRNAs in the diagnosis of acute ischemic stroke has not been clear. A literature review in 2018 aggregated reviewed data related to miRNA expression in 339 previous studies, involving 572 patients and 431 healthy controls in eight studies [7]. 22 miRNAs (12 up‐regulated and 10 down‐regulated) were reported as differential expression. In previous studies, differential expression analysis was used to identify the miRNAs differentially expressed after probe sequencing, but this method only focused on the role of a single gene, and could not find the relationship between genes and establish the relationship between genes and diseases afterward [8]. This problem can be solved by weighted gene co expression network analysis (WGCNA).

One of the obvious advantage is that WGCNA spread genes into co expression module, which provides a new system biology method based on microarray or RNA‐seq data, which is more often used to discover the relationship between network, gene and sample traits in a system with high sensitivity to low abundance, or small fold change gene without losing much information [9]. Previous studies have shown that WGCNA provides important modules and pathways for many diseases [10, 11, 12], and solved the problem in identifying miRNAs and up‐stream genes that play a key role in some diseases as well [13, 14].The purpose of this study is to identify is related pathways and genes based on the top 140 genes with high degrees by the method of WGCNA. This finding may help in detection and the biological function of stroke related genes.

## 2 MATERIAL AND METHODS

### 2.1 Study design and data preparation

**FIGURE 1:**
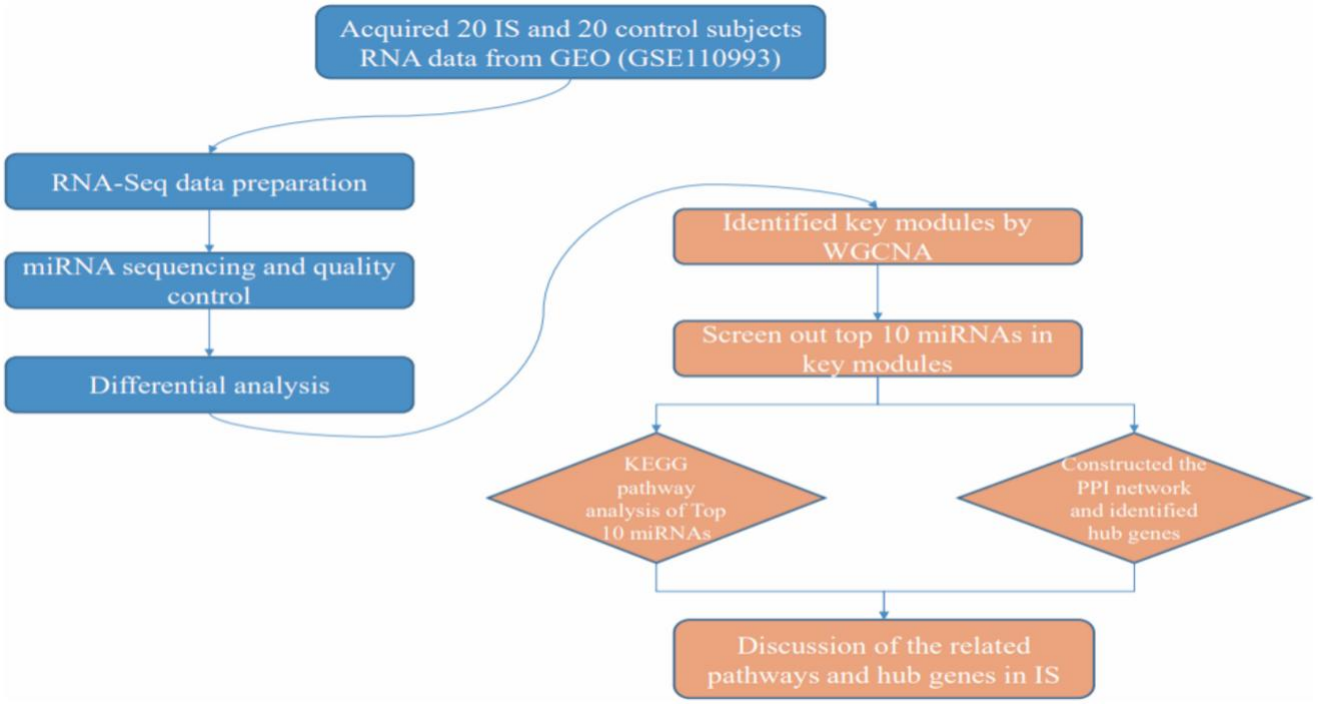
Flow chart diagram of study design showing the data preparation and analysis

The following flow chart diagram (Figure 1) with data preparation and data analysis showed the workflow of study design. In this study, IS gene expression data was downloaded from the public database of GEO. The most common type of sample in stroke is peripheral venous blood. From GEO, we obtained peripheral blood sequencing read counts from 20 IS and 20 healthy control patients.

Gene annotation was extracted by org.Hs.for example.db package with the corresponding information extracted including Entrez ID and gene symbols with reference to the.

Platform GPL570 (Illumina Inc.). To ensure the integrity and comparability of the data sets, normalization with log two transformation is calculated with RMA package [15]. For the identical gene symbol corresponding to multiple probes, the gene annotation is mapped with the probe with the highest average expression among all samples, and then the expression matrix of all samples is combined according to the miRNA/gene symbols. All samples were tested to remove batch effect using SVA toolkit, and standard Q‐Q diagram was drawn [16].

### 2.2 Quality control of raw data

The first step is to raw data processing. Samples sequencing read counts in SRA format is initially processed by internal Python script. In this step, clean data is obtained by deleting the too short read or read containing head adapter, the read containing deploy‐n tail, and excluding the low‐quality read(≤ 20%) from the original data [17]. In the meantime, the clean data of Q20, q30 and GC content were calculated. All downstream analyses are based on clean data of high quality.

### 2.3 Differential microRNA screening

40 samples of sequencing raw data (SRR6761159 ∼ SRR6761198) were obtained from GEO, and 60,617 count reads were obtained after header adapt and removing the redundant, after which, it was subsequently compared with the whole human genome (hg38). After eliminating the duplicate and abnormalities, the differential expressed genes were normalized and calculated with package DEseq, finally, genes with p. adjusted value < 0.05 and log2Foldchange > 1 were selected for the further analysis. This finding may be illustrative to elucidate their biological function in IS.

### 2.4 Construction of WGCNA network and identification of disease‐associated miRNAs

The data is processed with WGCNA package [18] in R Studio 3.6.0 software [19]. To ensure the reliability of network construction, abnormal genes were eliminated. First of all, the soft threshold of network construction is selected to make the adjacency matrix continuous between 0 and 1, so that the constructed network conforms to the power‐law distribution and is closer to the real biological network state [19]. Secondly, the scale‐free network is constructed by using the block module function, and then the module partition analysis is carried out to determine the gene co expression module, and the genes with similar expression patterns are paired and clustered [20]. By using the dynamic tree cutting algorithm, the cluster tree is cut into branches to define modules, and modules are assigned to different colors for visualization [21]. All modules are summarized by module eigengene (ME). Modules were characterized by its most important module eigengene (ME), which is calculated as a symbolic gene, representing the expression spectrum of all genes in a given module [22]. Module membership (MM) was defined as a correlation between individual gene and module eigengene [23]. In addition, Gene Significance (GS) of genes in the module is further calculated, which represents the correlation between genes and traits [24].

### 2.5 Identification of miRNA targets

The miRDB(http://mirdb.org/) database and the mirtarbase(http://mirtarbase.cuhk.edu.cn/) provides a large collection of predicted and experimentally verified miRNAs‐targets binding sites information. We downloaded the significant miRNA‐mRNA intersection data from the miRNAs‐target‐gene retrieval system, which contains all literature‐reported miRNA‐target genes. However, the validated target genes were from various diseases models, so the expression of those genes in IS might not be consistent. Then we selected the genes from the intersection between the two data and the significance miRNA‐targetgenes combination were defined.

### 2.6 GO enrichment analysis and KEGG pathway analysis

Based on the whole genome annotation information, we analyzed the Gene Ontology (GO) function using annotation, visualization and integrated discovery database (David) [25] and KOBAS 3.0 [26], and adjusted p‐value < 0.05. KEGG analysis was on KOBAS and further visualization was performed on the platform from ehbio (http://www.ehbio.com/). The 10 most‐enriched KEGG pathways are listed in the map and visualized with the online tools on the interactive Ehbio analysis platform.

### 2.7 PPI network construction

The STRING database (**Error! Hyperlink reference not valid.**) [27] has been applied to analysis the protein‐protein interactions (PPI) network. Confidence score > 0.4 was significant. In this study, we used R studio 3.5.0 to pick and visualize the target genes out of the first 140 hub‐genes using the 3.7.9 version of Cytoscape for visualization [28], and screened the hub genes in the network with reference to the correlation degree and biological interpretation. In this network, the degree can be recognized as the correlation with IS.

### 2.8 Cross validation

GSE22255 dataset is a previously released dataset of Ischemic stroke, which is obtained from the GEO database [29]. the gene expression was analyzed for peripheral blood monocytes from 20 IS patients and 20 age‐matched healthy control with Affymetrix oligonucleotide arrays. All 40 participants' nationality were Portuguese. The normalization with log2 fold change of GSE22255 were performed. Based on these data, the differential expression data in GSE22255 was analyzed by *T*‐test. *p*‐value and log 2‐fold change value were obtained separately. Then, GSE22255 was used to determine the expression changes of differential genes in RNA sequencing results of this study.

## 3 RESULTS

### 3.1 WGCNA network construction

**FIGURE 2:**
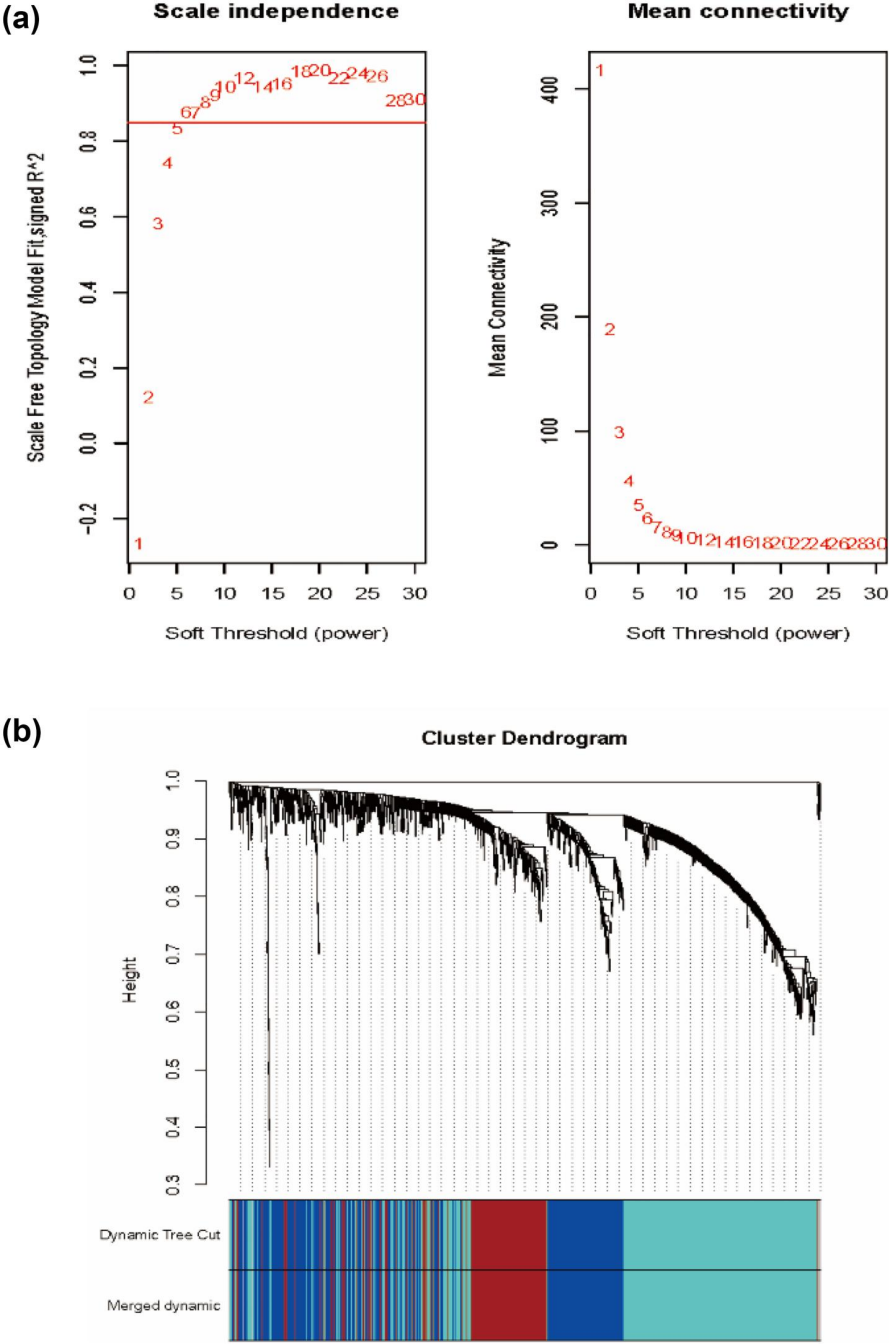
(a): Network topology analysis under different soft threshold power. The left panel shows the influence of soft threshold power on the scale‐free topological fit index; the right panel shows the influence of soft threshold power on the average connectivity. (b) Gene clustering tree (tree view) based on hierarchical clustering of adjacency differences

A total of 1544 miRNAs were screened by WGCNA. After removing the deleted and abnormalities, there are 294 screened differential expression miRNAs for subsequent analysis (|log2FoldChange| > 0.5, *p*‐value < 0.05). As shown in Figure 2A, when the soft threshold power is defined as 6, the scale‐free topological index is 0.9. Therefore, the network is closer to the real biological network state as it adheres to the power‐law distribution. The resulting gene tree and corresponding module colors are shown in Figure 2B. The number of genes per module is shown in supplementary Table S1.

### 3.2 Identification of clinical important modules

**FIGURE 3:**
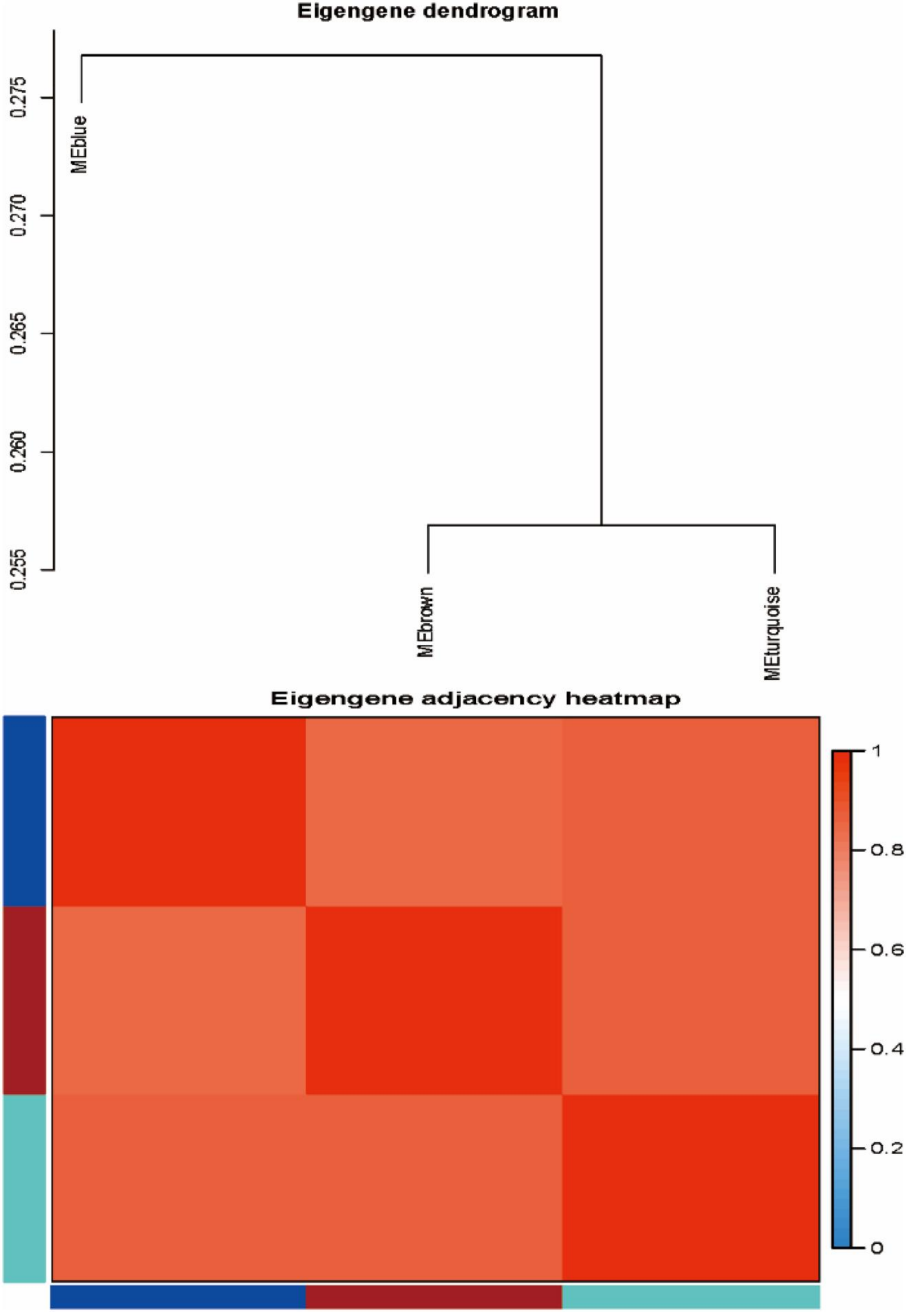
Heat map of traits and hub‐gene adjacency. The color bars on the left and below indicate the modules for each row or column

**FIGURE 4:**
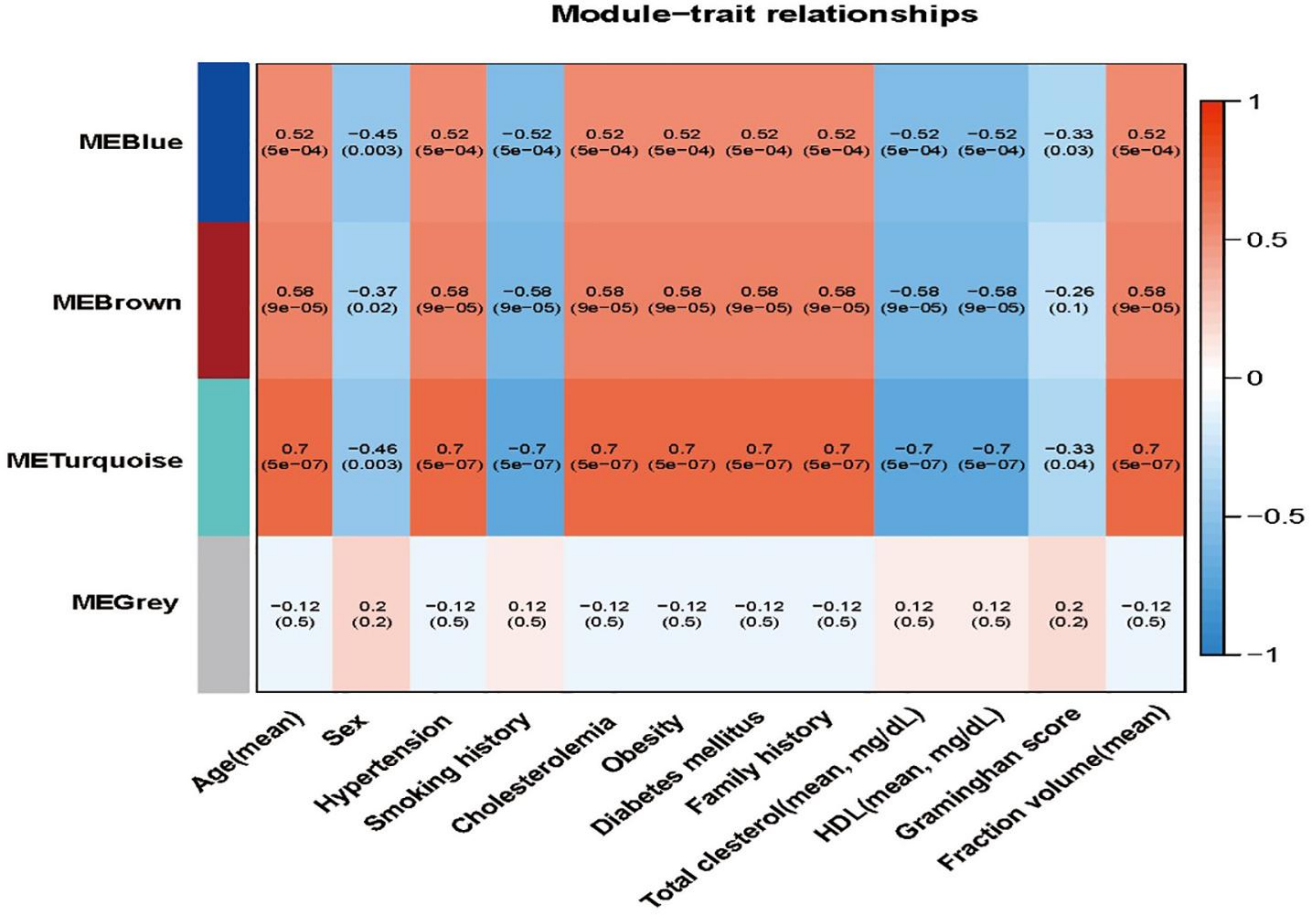
Module‐traits relationship. Each row corresponds to a module feature gene, and each column corresponds to a feature. Each cell contains the corresponding correlation and p‐value; red indicates positive correlation and blue indicates negative correlation

The heatmap shows the traits‐gene adjacency of the module (Figure 3). In this study, the parameters of 12 include age (mean), sex (M/F), hypertension (%), smoking history (%), hypercholesterolemia (%), obesity (%), diabetes mellitus (%), family history (%), total cholesterol (mean, mg/dl), HDL (mean, mg/dl) and infraction volume (mean). And on which basis, the Framingham score (%) to evaluate the risk of cardiovascular event is calculated. Our research focuses on the relationship of IS traits and gene type, so we initially focus on turquoise module(*r* = 0.7, *P* = 5e‐07), which has the highest correlation with the clinical traits of infraction volume in IS patients versus healthy control group (Figure 4).

### 3.3 Key miRNAs and miRNA‐target genes identification and functional annotation

The key miRNAs were considered to have high module membership (MM) and gene significance (GS), and were further selected for subsequent analysis (supplementary Table SA2). All the 11 miRNAs from the turquoise module were submitted, and by miRNA‐gene retrieval system, we got 2019 candidate genes of the 11 significant Key miRNAs (supplementary figure SA1). To verify this differential gene expression, we proceed to analyze the microarry data set from GSE162072 and differential gene expression analysis (|log2FoldChange| > 0.5, *p*‐value < 0.05) revealed significant change in 147 unique transcripts. The 125 differential expression and miRNA‐target genes common to the two datasets were used.

**FIGURE 5:**
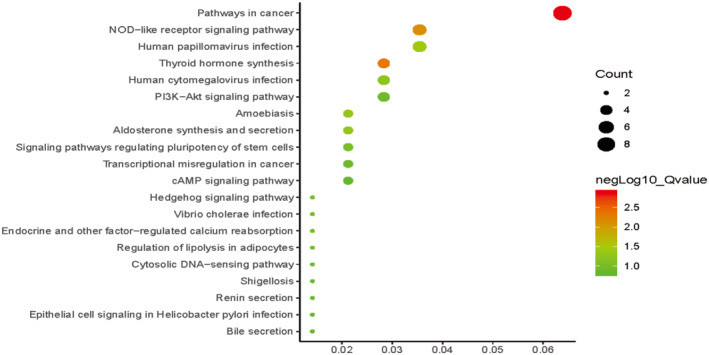
Kyoto Protocol Gene and Genome Encyclopedia pathway of hub‐genes in Ischemic Stroke. The *X* axis represents the enrichment gene ratio, and the *Y* axis represents the Kyoto Protocol Gene and Genome Encyclopedia term

Finally, David web tool was used to perform the representative Kyoto Encyclopedia gene and genome (KEGG) pathway to further clarify its function annotation. The two pathways are particularly important, in this study, the cancer pathway and gyroid hormone synthesis pathway were identified (Figure 5 and supplementary Table SA3).

### 3.4 PPI network

**FIGURE 6:**
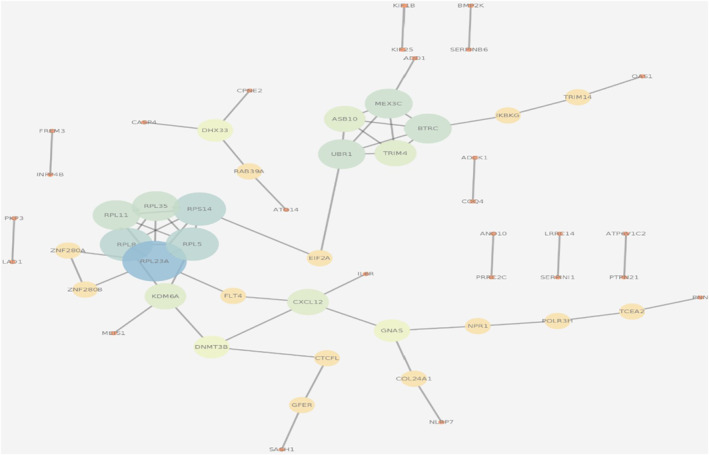
PPI network and hub genes. The hub gene was identified from the first 10 miRNAs by degree analysis. The depth of color indicates the level of key genes from low to high

The 125 differential expression and miRNA‐target genes were used to construct gene‐gene interactions using the STRING tool (https://string‐db.org/). There are 112 nodes and 43 edges in PPI network, representing protein‐protein interaction (Figure 6). The confidence level > 0.4 is set as criteria of significance. Combined with biological function interpretation, and selected top 5% degree genes, CXCL12 and EIF2A were assessed as hub genes (supplementary figure SA2).

### 3.5 Cross‐validation

The expression of differentially expressed genes was validated with GSE22255 dataset. RNA sequencing is better than microarray in characterizing transcriptions. However, the data of GSE22255 was measured by GPL570 (hgu133‐plus‐2) Affymetrix humangenomeu133 + 2.0 array. The expression profiles of differentially expressed genes and microRNAs are based on measured intensity of the array. Most of the probes in the array were mRNA, and a few belong to miRNA. Therefore, the miRNA probes in the array alone is not enough to cover all the genes in the study. The top 250 differentially expressed genes in GSE22255 with p‐value and log 2‐fold changes are shown in supplementary table SA4.

## 4 DISCUSSION

In this study, WGCNA method was used to study the pathways and target hub‐genes in IS. The pathways in cancer and Thyroid hormone synthesis signaling pathway and the first two genes of CXCL12 and EIF2A are considered to be biomarkers of important biological function in IS.

The CXCL12‐ CXCR4 axis formed by the interplay between CXCL12 and its specific receptor CXCR4, and the paired axis plays an important role in modulating the immunity and inflammation response, whose function is thought as regulating the development and function of hematopoietic system and lymphatic system, and participating in the development of central nervous system and tumor [30]. In recent years, it has been found that CXCL12 is closely related to the formation and stability of atherosclerosis plaque, and also plays an important role in angiogenesis, thrombosis and intimal hyperplasia in atherosclerotic lesions [31]. Multiple tumor associated signaling pathways are related with cell function, metabolism, growth, proliferation and survival, and also play an important role such as functional recovery of central nervous system injury, especially axon regeneration and autophagy [32]. Previous studies [33] have shown that ischemic stroke is a complex disease with cerebral ischemia, hypoxia necrosis and various other causes, resulting in the corresponding neurological deficit, in which inflammatory response is an important cause of post ischemic nerve damage, so inflammatory signal pathway has become the newly hot spotted for the treatment of ischemic stroke.

In recent years, many signaling pathways related to regulation of tumor are confirmed to be associated with cardiac disease, such as mTOR‐Akt signaling pathway [34], JAK‐STAT signaling pathway [35], as well as NF ‐ κ B (NF ‐ κ b) signaling pathway [36], which typically play a key role in the development of tumors related inflammatory response. However, recent studies showed the specific molecular mechanism of mir‐127‐5p inhibiting NF ‐ κ B signal pathway activity was further elucidated in cardiovascular disease [37]. It was reported that mir‐127‐5p can inhibit the phosphorylation level of p65 and affect its entry cell nucleus. Further study [38] showed that the mir‐127‐5p could inhibit the proliferation and clone formation of hepatoma cells, and also prohibit the expression of the target gene, biliverdin reductase B (BLVRB), by directly binding with 3′UTR.

In this study, combined with the results of KEGG and PPI network results, we found that CXCL12 plays a key role in the emergence and development of IS through two signaling pathways: pathways in cancer and Thyroid hormone synthesis signaling pathway, which may reveal that CXCL12 is a small but important molecular protein that can nest cells to circulate towards the injured niches, and itself is able to induce the progenitor cells to nest and migrate to the lesion site to promote the repairing and maintenance of homeostasis [39]. Chemokine CXCL12 and its specific receptor CXC chemokine receptor 4 (CXCR4) constitute the biological axis of CXCL12/CXCR4, which are involved in inflammatory reaction, tumor formation, and other disease [40]. This finding may reveal that CXCL12/CXCR4 plays an important role in the occurrence and development of cardiovascular and cerebrovascular diseases. What worthy of noting is that, miRNA‐16 is a newly discovered miRNA, which is involved in the progression of stroke by upregulating chemokine CXCL12 [41].

Protein kinase R‐like Er kinase (pErk) is a type I transmembrane protein located on the endoplasmic reticulum, which belongs to the upstream kinase family of EIF2a [42]. Endoplasmic reticulum stress perk‐eif2a signaling pathway, through the inhibition of protein synthesis to protect cells, promote cell survival [43]. With the extension of endoplasmic reticulum stress, the activation of *p*‐Erk‐EIF2a‐ATF4 signaling pathway can lead to the secretion of a variety of inflammatory factors, thus promoting the occurrence of inflammatory lesions [44]. In the meantime, it promotes apoptosis by inducing the expression of CHOP.

Further attempts to perform validation for the mechanism of these two pathways and key proteins may be valuable. However, this study has its limitations. The testing group is small with limited number of samples (40), given that WGCNA result is reliable only with the minimum sample number is 15, it is likely the result is reliable, besides, many genes in the identified module were reported to be closely related with IS, and one of the biomarker CXCL12 have been verified in another cohort. Although wet lab experiment is not tested in this study, we believe CXCL12 and EIF2a may be potential biomarker for accurate diagnosis and treatment of IS.

## 5 CONCLUSION

In conclusion, our study confirmed that pathways in cancer and gyroid hormone synthesis signaling pathway and two hub genes related to IS (CXCL12 and EIF2a) may be potential biomarker for accurate diagnosis and treatment of IS in future.

## Supporting information

Supporting information
