# Supplementary material for: Weighted gene co expression network analysis (WGCNA) with key pathways and hub‐genes related to micro RNAs in ischemic stroke
Source: IET Syst Biol. 2021 Apr 20;15(3):93–100. doi: 10.1049/syb2.12016 (PMC8675812; doi:10.1049/syb2.12016)
Supplement: Supplementary file 1 — Supporting information [file SYB2-15-93-s001.docx]

**
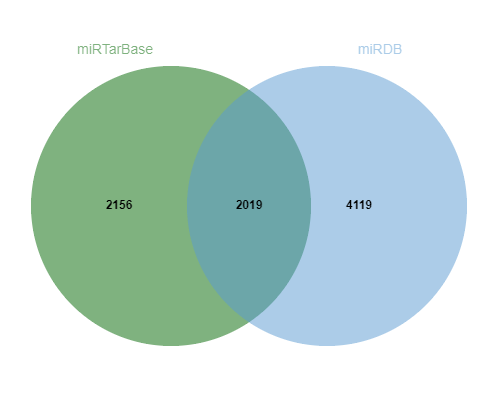
**

**supplementary figure S1 | MiRWalk 2.0-**

**validated-target miRNA-gene retrieval system, we got 2019 candidate genes of the 11 significant Key miRNAs by miTarBase and miRDB.**

**
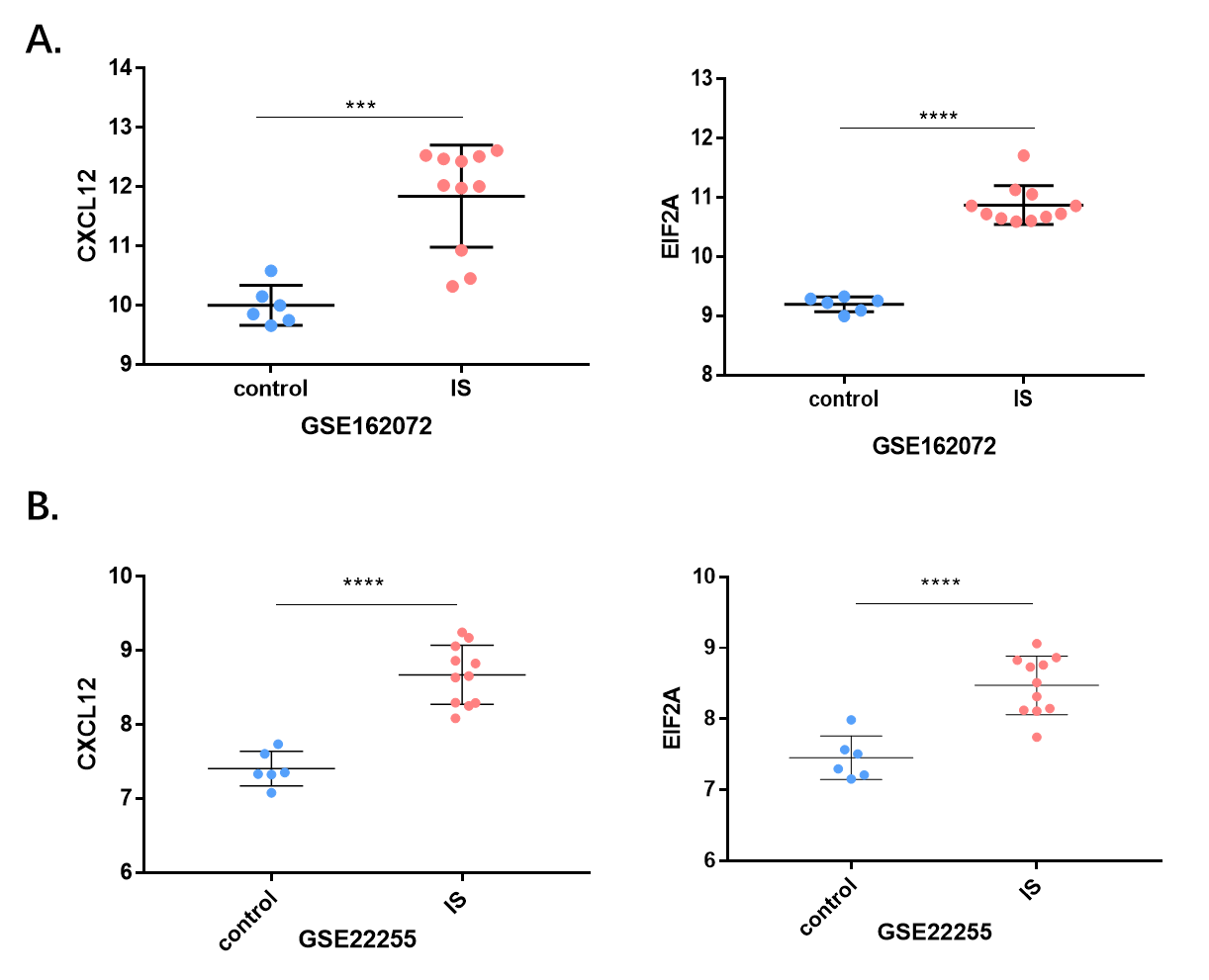
**

**supplementary figure S2 | Analyze the expression of CXCL12 and EIF2A from GSE162072(A) and GSE22255(B):** **red indicates high miRNA expression; blue indicates low miRNA expression.**
